# Fresh fruit consumption in relation to incident diabetes and diabetic vascular complications: A 7-y prospective study of 0.5 million Chinese adults

**DOI:** 10.1371/journal.pmed.1002279

**Published:** 2017-04-11

**Authors:** Huaidong Du, Liming Li, Derrick Bennett, Yu Guo, Iain Turnbull, Ling Yang, Fiona Bragg, Zheng Bian, Yiping Chen, Junshi Chen, Iona Y. Millwood, Sam Sansome, Liangcai Ma, Ying Huang, Ningmei Zhang, Xiangyang Zheng, Qiang Sun, Timothy J. Key, Rory Collins, Richard Peto, Zhengming Chen, China Kadoorie Biobank study

**Affiliations:** 1 Medical Research Council Population Health Research Unit, Nuffield Department of Population Health, University of Oxford, Oxford, United Kingdom; 2 Clinical Trial Service Unit and Epidemiological Studies Unit, Nuffield Department of Population Health, University of Oxford, Oxford, United Kingdom; 3 Department of Epidemiology and Biostatistics, School of Public Health, Peking University Health Science Center, Beijing, China; 4 Chinese Academy of Medical Sciences, Beijing, China; 5 China National Center for Food Safety Risk Assessment, Beijing, China; 6 Noncommunicable Disease Prevention and Control Department, Suzhou Center for Disease Control and Prevention, Suzhou, China; 7 Noncommunicable Disease Prevention and Control Department, Guangxi Center for Disease Control and Prevention, Nanning, China; 8 Noncommunicable Disease Prevention and Control Department, Sichuan Center for Disease Control and Prevention, Chengdu, China; 9 Noncommunicable Disease Prevention and Control Department, Meilan Center for Disease Control and Prevention, Haikou, China; 10 Pengzhou Center for Disease Control and Prevention, Pengzhou, China; 11 Cancer Epidemiology Unit, Nuffield Department of Population Health, University of Oxford, Oxford, United Kingdom; Stanford University, UNITED STATES

## Abstract

**Background:**

Despite the well-recognised health benefits of fresh fruit consumption, substantial uncertainties remain about its potential effects on incident diabetes and, among those with diabetes, on risks of death and major vascular complications.

**Methods and findings:**

Between June 2004 and July 2008, the nationwide China Kadoorie Biobank study recruited 0.5 million adults aged 30–79 (mean 51) y from ten diverse localities across China. During ~7 y of follow-up, 9,504 new diabetes cases were recorded among 482,591 participants without prevalent (previously diagnosed or screen-detected) diabetes at baseline, with an overall incidence rate of 2.8 per 1,000 person-years. Among 30,300 (5.9%) participants who had diabetes at baseline, 3,389 deaths occurred (overall mortality rate 16.5 per 1,000), along with 9,746 cases of macrovascular disease and 1,345 cases of microvascular disease. Cox regression yielded adjusted hazard ratios (HRs) associating each disease outcome with self-reported fresh fruit consumption, adjusting for potential confounders such as age, sex, region, socio-economic status, other lifestyle factors, body mass index, and family history of diabetes. Overall, 18.8% of participants reported consuming fresh fruit daily, and 6.4% never/rarely (non-consumers), with the proportion of non-consumers about three times higher in individuals with previously diagnosed diabetes (18.9%) than in those with screen-detected diabetes (6.7%) or no diabetes (6.0%). Among those without diabetes at baseline, higher fruit consumption was associated with significantly lower risk of developing diabetes (adjusted HR = 0.88 [95% CI 0.83–0.93] for daily versus non-consumers, *p <* 0.001, corresponding to a 0.2% difference in 5-y absolute risk), with a clear dose–response relationship. Among those with baseline diabetes, higher fruit consumption was associated with lower risks of all-cause mortality (adjusted HR = 0.83 [95% CI 0.74–0.93] per 100 g/d) and microvascular (0.72 [0.61–0.87]) and macrovascular (0.87 [0.82–0.93]) complications (*p <* 0.001), with similar HRs in individuals with previously diagnosed and screen-detected diabetes; estimated differences in 5-y absolute risk between daily and non-consumers were 1.9%, 1.1%, and 5.4%, respectively. The main limitation of this study was that, owing to its observational nature, we could not fully exclude the effects of residual confounding.

**Conclusion:**

In this large epidemiological study in Chinese adults, higher fresh fruit consumption was associated with significantly lower risk of diabetes and, among diabetic individuals, lower risks of death and development of major vascular complications.

## Abstract 2

Huaidong Du and colleagues study associations between fruit consumption and incidence of diabetes, along with complications of prevalent diabetes, in a Chinese population.

## Author summary

**Why was this study done?:**

- Worldwide evidence on the relevance of fruit consumption for the occurrence and progression of diabetes is still limited, although most dietary guidelines, including those for diabetes patients, recommend a higher level of fruit consumption.
- Although fruit and vegetables are often considered together, fruit may not be viewed as being as healthy as fresh vegetables for individuals with diabetes, given its relatively high sugar content. This has led to frequent abstention from fruit consumption among individuals with diabetes in many parts of the world (e.g., China).
- No prospective evidence has been available thus far showing the potential long-term impacts of fresh fruit consumption on the incidence of diabetes and, among individuals with diabetes, on the risks of diabetes-related vascular complications. Reliable findings on these two relationships within a single large cohort would be important for health promotion.

**What did the researchers do and find?:**

- We recruited over 500,000 Chinese adults from ten diverse areas across China during 2004–2008. Participants completed a detailed questionnaire interview and underwent physical measurements and blood tests, with their health tracked subsequently for seven years.
- Among individuals who were free of diabetes (either previously diagnosed or newly detected) at the start of the study, daily consumption of fresh fruit was associated with a 12% lower relative risk of developing diabetes, compared to never or rarely consuming fresh fruit.
- In those individuals who already had diabetes prior to the start of the study, consuming fresh fruit more than three days a week was associated with a 17% lower relative risk of dying from any cause and a 13%–28% lower risk of developing diabetes-related complications affecting large blood vessels (e.g., ischaemic heart disease and stroke) and small blood vessels (i.e., kidney diseases, eye diseases, and neuropathy) than those who consumed fruit less than one day per week.

**What do these findings mean?:**

- To our knowledge, this is the first large prospective study demonstrating similar inverse associations of fruit consumption with both incident diabetes and diabetic complications.
- These findings suggest that a higher intake of fresh fruit is potentially beneficial for primary and secondary prevention of diabetes.
- For individuals who have already developed diabetes, restricted consumption of fresh fruit, which is common in many parts of the world, e.g., China and other Asian countries, should not be encouraged.

## Introduction

Diabetes affects more than 400 million people globally, including about a quarter in China [1], with substantial risks of premature death and a range of macrovascular (e.g., ischaemic heart disease [IHD], stroke, and peripheral vascular disease) and microvascular (e.g., nephropathy, retinopathy, and neuropathy) complications. Healthy diet plays an important role in both prevention and appropriate management of diabetes [2], and diets rich in fruit and vegetables are generally recommended [3,4], even though evidence about their effects, particularly for fruit consumption, among diabetic patients is still rather limited.

Fruit and vegetables share many common nutritional properties, but are often consumed in different settings and manners, especially in China, where fresh fruit is usually consumed raw as a snack while fresh vegetables are usually fried or stewed (often together with meat, cooking oil, and salt) as main dishes. Moreover, the sugar content in fruit is generally higher than in vegetables, leading to concerns about its potential harmful impacts on diabetes [5]. A few prospective studies have tried to assess the effects of fruit intake on risk of diabetes, but the results have been inconsistent, with some studies showing a moderately strong inverse association [6,7] and others, including those in China [8] and in European populations [9], finding no association. Furthermore, there is very limited evidence about the effects of fruit consumption on risks of death and major vascular complications among people with established diabetes [10–12]. Reliable assessment of the effects of fruit consumption on risks of incident diabetes and, among those who have already developed diabetes, on diabetic complications is urgently needed to improve dietary recommendations, especially in low- and middle-income countries such as China and other Asian countries where avoidance of sweet-tasting food (including fresh fruit) is common among diabetic patients [13–15].

With data from the China Kadoorie Biobank study, a prospective cohort study of 0.5 million adults, we examined the association of fresh fruit consumption with the risk of developing diabetes among people without baseline diabetes, and with the risks of death and major vascular hospitalisations among people with prevalent diabetes at baseline.

## Methods

The China Kadoorie Biobank study was conducted in accordance with a predefined study protocol [16,17], and data analyses were performed following a prespecified analysis plan (S1 Text).

### Ethics statement

Ethics approval was obtained from the Oxford University Tropical Research Ethics Committee, the Chinese Academy of Medical Sciences Ethical Review Committee, the Chinese Center for Disease Control and Prevention Ethical Review Committee, and the scientific review boards in each of the ten regional centres.

### Study population

The China Kadoorie Biobank study is a large nationwide prospective cohort study involving ten geographically diverse regional sites (five urban and five rural) in China, chosen to cover a wide range of risk exposures and disease patterns, all with good-quality death and disease registries and local capacity. The study design, methods, and population have been reported previously [16,17]. In brief, between June 2004 and July 2008, all non-disabled permanent residents within preselected communities aged 35 to 74 y were invited to participate in the study, and about one in three (33% in rural areas, 27% in urban areas) responded, yielding a total of 512,891 participants (including a few who were just outside the targeted age range); all participants provided written informed consent.

### Data collection

At the local study clinics, trained health workers administered a laptop-based questionnaire on socio-economic status, smoking, alcohol intake, diet, physical activity [18], and medical history; measured anthropometrics and blood pressure; and took 10 ml of venous blood for on-site testing of random blood glucose (RBG) (with time since last eating or drinking any energy-containing foods or beverages recorded) and for long-term storage. RBG level was measured immediately following sample collection using the SureStep Plus System (Johnson & Johnson), which provided plasma-equivalent readings and was regularly calibrated with manufacturer control solutions. Individuals with no prior history of physician-diagnosed diabetes but with RBG levels between 7.8 and 11.1 mmol/l were invited back the following day for a fasting blood glucose test. At baseline, individuals were considered as having prevalent diabetes if they had either a self-reported prior history of physician-diagnosed diabetes or screen-detected diabetes, which was defined as having never been diagnosed with diabetes but having a measured RBG level ≥7.0 mmol/l with time since last food/beverages ≥8 h, or ≥11.1 mmol/l with time since last food/beverages <8 h, or a fasting blood glucose level ≥7.0 mmol/l on subsequent testing [19]. Dietary data covered 12 major food groups, including fresh fruit, fresh and preserved vegetables, meat, and dairy products, each with five frequency levels about habitual consumption during the past 12 mo (daily, 4–6 d/wk, 1–3 d/wk, monthly, or never/rarely) [20].

Following the baseline survey, 5%–6% of the surviving participants were randomly selected for resurveys in 2008 (first resurvey, response rate 80%) and 2013–2014 (second resurvey, response rate 76%), using procedures similar to those at baseline. During the second resurvey, in addition to frequency, the quantity of each food group consumed was also collected, which was used as a proxy to estimate the group average consumption for each baseline category (S1 Table).

### Follow-up for mortality and morbidity

All participants were followed up (2,411 [0.5%] participants were lost to follow-up by 1 January 2014) for death and disease using information collected through linkages with death and disease registries and health insurance databases. The vital status of each participant was obtained periodically through China’s Disease Surveillance Points system [21], checked annually against local residential and health insurance records, and by street committees or village administrators. In addition, information on any episodes of hospitalisation was collected through linkages with disease registries (for stroke, IHD, cancer, and diabetes) and national health insurance claim databases. Cause-specific deaths and non-fatal events were coded, blinded to baseline information, by the trained staff using ICD-10 [16]. In addition, diagnosis descriptions of diabetes-related events (both fatal and non-fatal) were reviewed and standardised centrally by study clinicians blinded to baseline fruit consumption. For the present study, incident diabetes included all reported cases (fatal or not) of new onset diabetes that occurred between ages 35 and 79 y. Underlying causes of death were classified as diabetes, cardiovascular disease (CVD), or other. Diabetes-related microvascular complications included nephropathy, retinopathy, and neuropathy. Macrovascular complications included IHD, stroke, and other (S2 Table). Only the first non-fatal event for each endpoint was considered.

### Statistical analysis

For baseline characteristics, means (standard deviations) or percentages of individuals by diabetes status were calculated, adjusting for age, sex, and region, where appropriate, using either multiple linear (for continuous outcomes) or logistic regression (for binary outcomes).

Among the 482,591 participants who were free of diabetes at baseline, hazard ratios (HRs) and 95% CIs for diabetes incidence by fresh fruit consumption level were estimated using Cox proportional hazard regression. Analyses were stratified by age at risk, sex, and region and were adjusted for education (four categories), annual household income (four categories), smoking (four categories), alcohol intake (four categories), physical activity (continuous variable), body mass index (BMI) (continuous variable), survey season (four categories), family history of diabetes (dichotomous), and consumption of meat (three categories), dairy products (three categories), and preserved vegetables (five categories).

Among those 30,300 participants with diabetes at baseline, Cox regression was used to investigate the association of fruit consumption with hospitalisations due to different diabetes complications. The main analyses grouped participants into three categories of fruit consumption (<1 d/wk, 1–3 d/wk, or >3 d/wk) in order to retain a reasonable number of cases in each group. In addition to the covariates mentioned above, baseline CVD and diabetes status (both as dichotomous variables) and anti-diabetic treatment (four categories) were also adjusted for.

Separate analyses of the first and second halves of the follow-up period showed no clear deviation from the proportionality assumption. The floating absolute risk method was used to calculate the confidence intervals of HRs in all categories (including the reference category) of fruit consumption. The traditional approach with an arbitrarily chosen reference group is unsatisfactory as the standard errors and associated CIs are dependent on the precision within the reference group; therefore, comparisons can only be made with the reference group. In contrast, the floating absolute risk method estimates standard errors and CIs using “floated” variances to provide appropriate variances to the log relative risk (i.e., HR in our analyses) for all exposure categories, including the reference category. Hence, valid comparisons can be made between any two exposure groups for polychotomous risk factors [22,23]. To further quantify the linear association between fruit consumption amount and disease risks and to account for regression dilution bias [24,25], we used data from the two resurveys to estimate mean usual fruit intake (portions/month) for each baseline category (S1 Table). The group mean levels of usual consumption were used to plot against the HRs in each baseline exposure category and to yield the effect size per one daily portion (i.e., 100 g/d) of usual fruit consumption through the Cox regression analyses.

Adjusted HRs for each one daily portion of usual fruit consumption were calculated across strata of potential effect modifiers, e.g., factors related to diabetes risk and diabetes stage, and chi-square tests for trend and heterogeneity were applied to the log HRs and their standard errors. In addition, sensitivity analyses investigated the potential impacts of excluding the first 2 y of follow-up, excluding participants with prevalent CVD at baseline, and additional adjustment for other dietary factors. For analyses of diabetes incidence, sensitivity analysis was also performed excluding participants with incident CVD (i.e., myocardial infarction and stroke) during follow-up.

All analyses were conducted in SAS (version 9.2), and graphs were plotted in R 3.0.2.

## Results

Of the 512,891 participants, 30,300 (5.9%) had diabetes at baseline, including 16,162 with previously diagnosed diabetes and 14,138 with screen-detected diabetes (Table 1). Based on age at diagnosis being <30 y and insulin use, 0.2% of the cases were likely to be type 1 diabetes. Individuals with diabetes were older and were more likely to be women, to live in urban areas, to be less physically active, and to have higher levels of BMI, waist circumference, and blood pressure. Among men, the proportions with current regular smoking and alcohol intake were about 10% lower in those with previously diagnosed diabetes than in those with screen-detected diabetes or those without diabetes.

**Table 1:** Selected characteristics of participants by baseline diabetes status.

Characteristic	No diabetes (*n* = 482,591)	Screen-detected diabetes (*n* = 14,138)	Previously diagnosed diabetes (*n* = 16,162)	Overall (*n* = 512,891)
**Age, years**	51.2 (10.5)	56.0 (10.5)	58.8 (10.5)	51.5 (10.7)
**Women**	58.8	61.9	64.3	59.0
**Urban resident**	43.1	54.8	63.6	44.1
**Education > 6 y**	49.2	45.7	52.1	49.2
**Household income > 20,000 yuan/year**	42.6	43.3	45.9	42.7
**Current smoking**				
Men	61.4	60.5	51.4	61.1
Women	2.4	2.3	2.1	2.4
**Current alcohol intake**				
Men	33.6	35.7	21.2	14.8
Women	2.1	1.6	0.7	2.1
**Physical activity, MET-h/d**	21.2 (11.9)	19.8 (11.9)	18.5 (12.0)	21.1 (13.9)
**BMI, kg/m**^2^*	23.6 (3.2)	25.0 (3.2)	24.7 (3.3)	23.7 (3.4)
**Waist circumference, cm**	80.0 (9.2)	85.1 (9.2)	84.6 (9.3)	80.3 (9.8)
**SBP, mm Hg**	130.6 (19.6)	139.2 (19.6)	137.8 (19.8)	131.1 (21.3)
Random blood glucose, mmol/l*	5.7 (1.7)	13.0 (1.7)	11.2 (1.7)	6.1 (2.3)
**Prior IHD**	2.9	3.0	6.7	3.0
**Prior stroke**	1.6	2.2	4.0	1.7
**Family history of diabetes**	6.4	12.1	24.8	7.1
**Regular food consumption**^†^				
Meat	47.1	47.9	49.6	47.2
Dairy products	11.7	10.6	20.3	11.9
Preserved vegetables	22.7	22.7	20.4	22.6
Fresh vegetables	94.8	94.6	95.7	94.8
**Fresh fruit consumption**				
Never/rarely	6.0	6.7	18.9	6.4
Monthly	33.9	35.0	34.7	34.0
1–3 d/wk	31.6	31.3	26.6	31.5
4–6 d/wk	9.4	9.8	7.3	9.4
Daily	19.1	17.2	12.5	18.8

Values are mean (standard deviation) or percentage and were adjusted for age, sex, and region, where appropriate.

*Random blood glucose was missing for 8,341 participants. BMI was missing for two participants.

^**†**^Regular consumption means consuming food products for at least 4 d/wk, except for fresh vegetables, where it means daily consumption.

BMI, body mass index; IHD, ischaemic heart disease; MET, metabolic equivalent; SBP, systolic blood pressure.

Overall, 18.8% of participants reported consuming fresh fruit daily (daily consumers) and 6.4% never or rarely (non-consumers). The proportion of non-consumers among those with previously diagnosed diabetes (18.9%) was about three times higher than among those with screen-detected diabetes (6.7%) and those without diabetes (6.0%). There was an overall weak inverse association of fruit consumption with blood glucose (RBG was 0.6 mmol/l lower among daily consumers than non-consumers; S3 Table). A similar inverse association was seen among those with previously diagnosed diabetes, even after additionally adjusting for all other potential confounders including fasting time and anti-diabetic medications (S4 Table).

**Fig 1:**
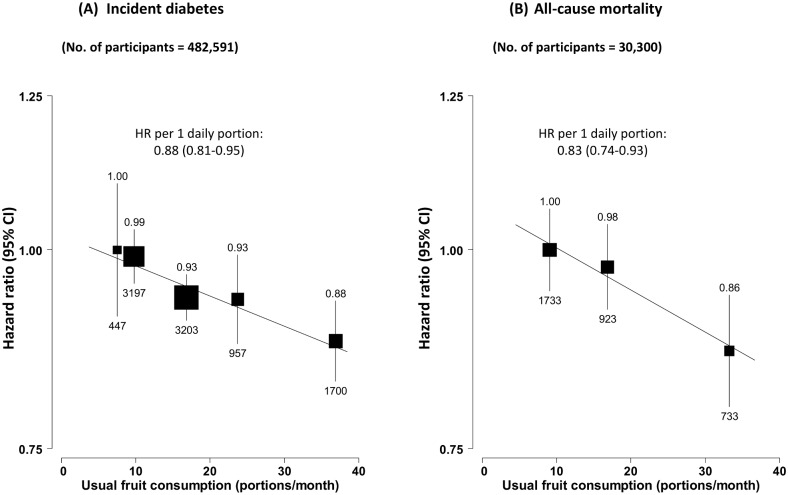
Adjusted hazard ratios for incident diabetes and all-cause mortality among those with diabetes at baseline, by fresh fruit consumption. (A) Incident diabetes; (B) all-cause mortality among those with diabetes at baseline. Analyses were stratified by age at risk, sex, and region and were adjusted for education, income, alcohol intake, smoking, physical activity, survey season, BMI, family history of diabetes, and intakes of dairy products, meat, and preserved vegetables. The black boxes represent the hazard ratios (HRs), with the size inversely proportional to the variance of the log HRs, and the vertical lines represent the 95% confidence intervals. The values above the vertical lines are the point estimates of the HRs, and the values below them are the numbers of cases.

During ~7 y (3.4 million person-years) of follow-up, 9,504 new onset cases of diabetes were recorded among the 482,591 participants without diabetes at baseline, with an overall incidence rate of 2.8 per 1,000 person-years (Table 2). Participants with higher fruit consumption had a significantly lower risk of developing diabetes, with the adjusted HR for daily consumers versus non-consumers being 0.88 (95% CI 0.83–0.93) (Fig 1A). After adjusting for regression dilution bias, there was a clear log-linear dose–response relationship, with each one daily portion of fruit associated with an adjusted HR of 0.88 (95% CI 0.81–0.95) (*p* for trend = 0.01). This association was not significantly modified by sex, age, region, survey season, or a range of other factors including smoking, alcohol consumption, physical activity, BMI, and family history of diabetes (*p* for trend or heterogeneity ≥ 0.2 for all; S1 Fig). Excluding baseline or incident IHD and stroke, additionally adjusting for other dietary variables, or excluding the first 2 y of follow-up did not materially alter the results (S5 Table).

**Table 2:** Incidence rate and 5-y risk of incident diabetes and, among those with prevalent diabetes at baseline, diabetes complications by level of fresh fruit consumption.

Measure	New diabetes onset in those without diabetes at baseline (*n =* 482,591)	Events in people with diabetes at baseline (*n =* 30,300)		
		Death from any cause	Microvascular complications	Macrovascular complications
**Overall incidence rate, per 1,000 person-years**	2.8	16.5	6.7	55.9
5-y risk by level of fresh fruit consumption, percent*				
Never/rarely (non-consumers)	1.5	9.2	3.9	30.0
Monthly	1.5	8.2	3.6	28.6
1–3 d/wk	1.4	8.4	3.2	28.2
4–6 d/wk	1.4	7.7	2.7	27.2
Daily	1.3	7.3	2.8	24.6

*Estimated from rates calculated by multiplying the fully adjusted hazard ratios in each of the five groups (presented in Fig 1 and Table 3) by a common multiple that was chosen so that the weighted mean rate in all five groups matched the overall rate in the whole study.

**Fig 2:**
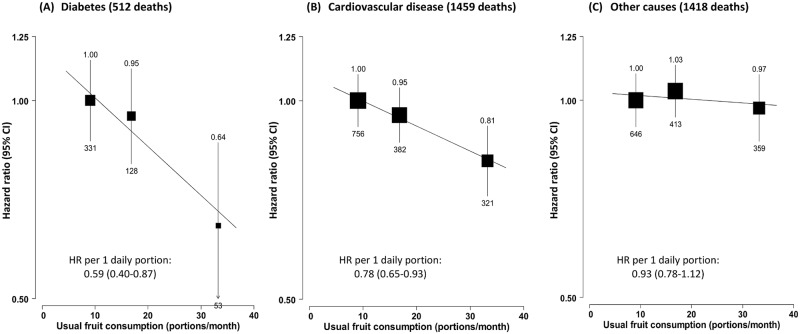
Adjusted hazard ratios for selected cause-specific mortality by fresh fruit consumption among 30,300 participants with diabetes at baseline. Mortality from (A) diabetes, (B) cardiovascular disease, and (C) other causes. Conventions as in Fig 1. Baseline status for cardiovascular disease, diabetes, and anti-diabetic treatment were also adjusted for. HR, hazard ratio.

Among the 30,300 participants who had prevalent diabetes at baseline, 3,389 (11.2%) died during follow-up (overall mortality rate 16.5 per 1,000), including 1,459 (43.1%) from CVD, 512 (15.1%) from diabetes (i.e., acute diabetic crises or other unspecified diabetes deaths without any immediate or antecedent cause of death, e.g., vascular or renal cause of death), and 1,418 (41.8%) from other causes, including cancer (*n =* 790). Fruit consumption was significantly and inversely associated with mortality from all causes (Fig 1B), diabetes, and CVD, but not with mortality from other causes (including cancer) (Fig 2). Compared to those who consumed fresh fruit <1 d/wk, individuals who consumed fruit >3 d/wk had adjusted HRs of 0.86 (95% CI 0.80–0.94) for all-cause mortality, 0.64 (0.48–0.86) for diabetes mortality, and 0.81 (0.72–0.92) for CVD mortality. These associations were approximately log-linear after correcting for regression dilution bias, with each one daily portion of fruit associated with HRs of 0.83 (95% CI 0.74–0.93), 0.59 (0.40–0.87), and 0.78 (0.65–0.93), respectively.

**Fig 3:**
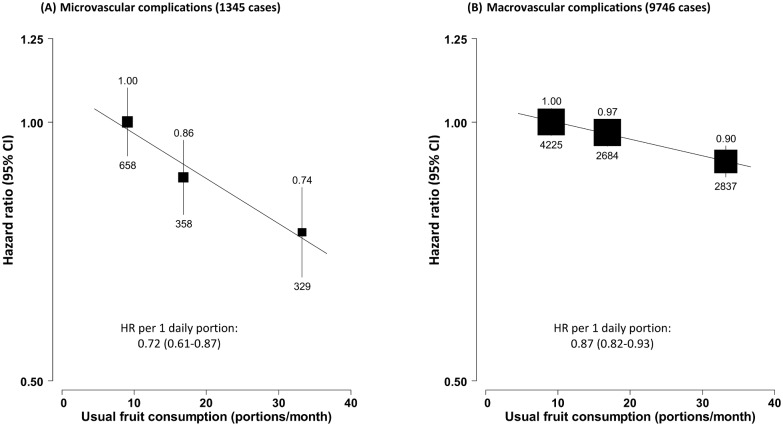
Adjusted hazard ratios for macro- and microvascular complications of diabetes by fresh fruit consumption among 30,300 participants with diabetes at baseline. (A) Microvascular complications; (B) macrovascular complications. Conventions as in Fig 1. HR, hazard ratio.

**Fig 4:**
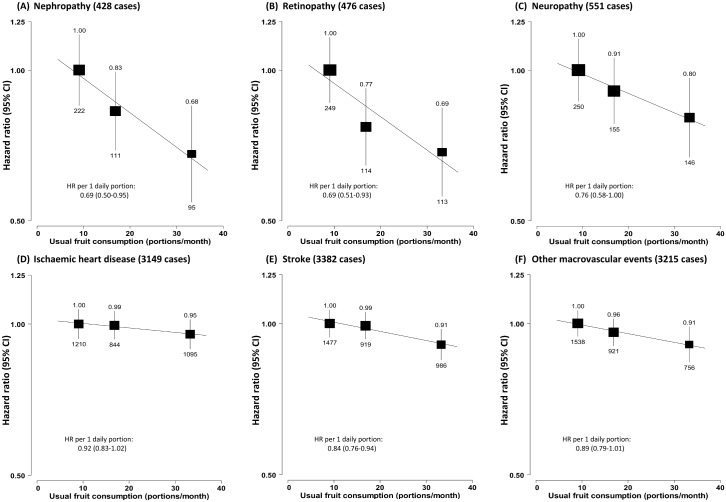
Adjusted hazard ratios for major diabetes-related vascular complications by fresh fruit consumption among 30,300 participants with diabetes at baseline. (A) Nephropathy; (B) retinopathy; (C) neuropathy; (D) ischaemic heart disease; (E) stroke; (F) other macrovascular events. Conventions as in Fig 1. HR, hazard ratio.

Fruit consumption was also inversely associated with risk of hospitalisation due to diabetic vascular complications. Compared to consuming fruit <1 d/wk, consuming fruit >3 d/wk was associated with a 26% (95% CI 16%–34%) lower risk of microvascular complications and 10% (6%–14%) lower risk of macrovascular complications. Each daily portion was associated with HRs of 0.72 (95% CI 0.61–0.87) and 0.87 (0.82–0.93), respectively (Fig 3). The strength of the association of fruit consumption with individual complications was similar (Fig 4).

The strength of the associations between fruit consumption and diabetic complications were largely consistent across subgroups of participants classified by baseline characteristics, survey season, baseline RBG level, and diabetes status (previously diagnosed versus screen-detected) (*p* for trend or heterogeneity ≥ 0.1 for all; S2 Fig). Anti-diabetic treatment, age of diabetes onset, and diabetes duration did not seem to modify the associations either (S3 Fig).

Similar findings were observed when fresh fruit consumption was analysed in the original five categories, although the trend was somewhat less consistent due to the smaller number of cases in each group (Table 3). Additional adjustment for other dietary variables, excluding the first 2 y of follow-up, or excluding diabetic participants with prevalent IHD or stroke at baseline (*n =* 3,885) did not materially alter the observed associations (S5 Table).

**Table 3:** Number of events and adjusted hazard ratios (95% CIs) for diabetic complications according to five categories of fresh fruit consumption.

Outcome	Frequency of fresh fruit consumption					*p*_trend_
	Never/rarely	Monthly	1–3 d/wk	4–6 d/wk	Daily	
**All-cause mortality**						
Number of events	643	1,090	923	201	532	
HR	1.00	0.90	0.91	0.84	0.79	0.002
95% CI	0.92–1.09	0.84–0.96	0.85–0.97	0.73–0.96	0.72–0.87	
**Diabetes mortality**						
Number of events	150	181	128	17	36	
HR	1.0	0.74	0.79	0.51	0.57	0.008
95% CI	0.84–1.19	0.63–0.87	0.66–0.95	0.32–0.83	0.40–0.81	
**Cardiovascular mortality**						
Number of events	265	491	382	82	239	
HR	1.00	1.02	0.96	0.86	0.81	0.006
95% CI	0.88–1.14	0.93–1.13	0.87–1.07	0.69–1.08	0.70–0.93	
**Total macrovasuclar complications**						
Number of events	1,383	2,842	2,684	717	2,120	
HR	1.00	0.95	0.94	0.91	0.86	<0.001
95% CI	0.95–1.06	0.91–0.99	0.90–0.98	0.84–0.98	0.82–0.90	
**Ischaemic heart disease**						
Number of events	473	737	844	222	873	
HR	1.00	0.89	0.92	0.92	0.88	0.19
95% CI	0.91–1.10	0.82–0.96	0.86–0.98	0.81–1.05	0.82–0.95	
**Stroke**						
Number of events	530	947	919	271	715	
HR	1.00	0.96	0.96	1.02	0.84	0.004
95% CI	0.91–1.10	0.90–1.04	0.90–1.03	0.90–1.15	0.77–0.91	
**Other macrovascular events**						
Number of events	380	1,158	921	224	532	
HR	1.00	1.00	0.96	0.85	0.94	0.17
95% CI	0.90–1.11	0.93–1.06	0.90–1.02	0.75–0.97	0.85–1.04	
**Total microvascular complications**						
Number of events	234	424	358	78	251	
HR	1.00	0.93	0.82	0.69	0.72	<0.001
95% CI	0.87–1.15	0.84–1.04	0.74–0.91	0.55–0.87	0.63–0.83	
**Nephropathy**						
Number of events	89	133	111	17	78	
HR	1.00	0.89	0.77	0.49	0.69	0.02
95% CI	0.80–1.25	0.74–1.08	0.64–0.93	0.30–0.79	0.53–0.88	
**Retinopathy**						
Number of events	83	166	114	23	90	
HR	1.00	1.12	0.83	0.67	0.76	0.01
95% CI	0.79–1.26	0.95–1.33	0.69–1.00	0.44–1.02	0.60–0.96	
**Neuropathy**						
Number of events	83	167	155	41	105	
HR	1.00	0.86	0.82	0.77	0.71	0.05
95% CI	0.73–1.03	0.70–0.96	0.70–0.96	0.56–1.05	0.57–0.88	

Analyses were stratified by age at risk, sex, and region and adjusted for education, income, alcohol intake, smoking, physical activity, survey season, body mass index, family history of diabetes, baseline status of diabetes and cardiovascular disease, anti-diabetic treatment, and intakes of dairy products, meat and preserved vegetables. HR, hazard ratio.

## Discussion

This large prospective study of Chinese adults with and without diabetes showed that higher fresh fruit consumption was significantly associated with a lower risk of developing diabetes, and also with a lower risk of dying or developing vascular complications among those who have already developed diabetes. These associations appeared to be similar in both men and women, in urban and rural residents, and in those with previously diagnosed and screen-detected diabetes. Moreover, higher fresh fruit consumption was not associated with elevated level of blood glucose.

Several prospective studies have previously assessed the association of fruit consumption with risk of diabetes, showing inconsistent findings [6,7,9,26–29]. For example, higher fruit consumption was significantly associated with diabetes incidence in the Nurses’ Health Study (with 6,358 cases) and a small Finnish study with 383 cases [6,30], with HRs of 0.82 (95% CI 0.72–0.92) and 0.69 (0.50–0.93), respectively, when comparing the highest with the lowest fruit consumption category. No significant association, however, was observed in the European Prospective Investigation into Cancer and Nutrition (EPIC)–InterAct study with nearly 11,000 incident diabetes cases [9] or a cohort study of Chinese women with approximately 1,600 new diabetes cases [8]. In the most recent meta-analysis of >400,000 participants from 11 studies with nearly 34,000 incident diabetes cases, higher fruit consumption was associated with a 9% (95% CI 4%–13%) lower risk of diabetes incidence [27]. These previous studies were conducted primarily among Western populations and tended to combine fresh fruit with processed fruit (sometimes including also fruit juice), in contrast to focusing only on fresh fruit, as in our study. This may partly explain the much stronger linear association observed in our study. In addition, the stronger association we observed might also be due to the very low level of fruit consumption among Chinese people and a non-linear dose–response association between fruit intake and diabetes risk [7,29]. Furthermore, our study has taken into account regression dilution bias when estimating the linear associations [24,25] while few previous large prospective studies have dealt with this important issue [31].

To date, very few large prospective studies have assessed the long-term health effects of fruit consumption in people with diabetes, even though fruit has been rather consistently associated with lower risk of CVD [20], a major complication of diabetes [19]. In two reports from the EPIC study, one involving approximately 6,000 [12] and the other involving 10,500 [11] individuals with self-reported diabetes, one portion of fruit per day was significantly associated with 12%–15% lower all-cause [12] and cardiovascular mortality [11]. Only one small study in Japan has reported the association of fruit consumption with diabetes-related microvascular complications. In that study of nearly 1,000 diabetic patients with 8 y of follow-up, individuals in the highest quartile of fruit consumption had approximately 50% (HR = 0.48 [95% CI 0.32–0.71]) lower risk of developing diabetic retinopathy (total 285 cases) [10]. Our study included a much larger number of participants and well-characterised disease outcomes, with new findings about the potential benefits of fresh fruit intake on a range of macro- and microvascular complications of diabetes. In addition, a separate analysis of data from more than 70,000 participants with existing CVD or hypertension in the China Kadoorie Biobank, with an overlap of about 10,000 participants with the current study, showed similar inverse associations of fresh fruit consumption with all-cause and cardiovascular mortality [32].

This is the first cohort study to our knowledge reporting beneficial associations of fresh fruit consumption with both incidence of diabetes and development of diabetes complications. Given the large sample size, our study findings are statistically robust even though the causality of the association cannot be established from such observational studies. However, the study also has limitations. First, our dietary questionnaire was not validated against another reference method, and information on fruit types was not collected. However, the previously observed inverse associations of fruit consumption with blood pressure and risk of CVD [20] could indicate an appropriate predictive validity of this method in estimating fruit consumption [33]. Moreover, the baseline level and secular trend of fruit consumption observed in our study population were in line with the findings from nationally representative nutrition surveys that used three consecutive 24-h recalls to collect habitual intake [20]. Fruits with relatively lower glycaemic index (i.e., apples, oranges, pears, and berries) may have larger beneficial effects on diabetes than those with a higher glycaemic index (i.e., bananas, grapes, and tropical fruits) [34], although previous studies have found both higher and lower glycaemic index fruits to be associated with lower risk of diabetes [6]. Based on data from the China Health and Nutrition Survey, the most frequently consumed fruits in China are apples, pears, and oranges, which are temperate climate/low glycaemic index fruit. Second, our baseline dietary data included consumption frequency only (not amount); therefore, the linear associations (i.e., HRs per 100 g/d) were estimated based on assumptions (assuming daily portions of fruit consumption did not change from baseline to second resurvey). Also, our analyses could not be adjusted for total energy or specific nutrient intake, and our data do not allow a reliable assessment of how much fruit per day is too much. Other dietary factors that may have an important association with diabetes, e.g., sugar-sweetened beverages [35], could not be adjusted for due to lack of information. This, however, should not confound our findings because the average sugar-sweetened beverage consumption level in our population was very low [36]. Third, no information on vascular diseases other than IHD and stroke was collected at baseline; thus, some cases of diabetes complications might be recurrent instead of new onset. This again should not invalidate our findings, given the very similar associations observed in individuals with newly detected diabetes (who should be less likely to have diabetes complications at baseline) and previously diagnosed diabetes. A proportion of diabetes cases might not have been detected at baseline because we did not use post-load blood glucose and HbA1c tests at baseline. Such misclassification should be non-differential (i.e., the proportion of undiagnosed diabetes cases should not be related to the level of fresh fruit consumption) and therefore should not invalidate or overestimate the observed associations of fruit consumption with diabetes incidence and vascular complications. Finally, although we carefully adjusted for potential confounders and there were consistent results across different participant subgroups, residual confounding (e.g., by socio-economic status) may still persist. In other words, it is not possible to determine reliably from this study whether the somewhat lower risks of diabetes incidence and diabetes complications observed among those with higher fruit consumption were caused by fruit consumption per se or were mainly due to other factors.

The exact mechanisms through which fresh fruit consumption may be protective against the development and deterioration of diabetes are not very well understood. Fruit contains sugars (i.e., glucose and fructose), which may have negative impacts on glycaemic control [37]. However, the natural sugars in fruit may not be metabolised in the same way as refined sugars [38]. In our study, fruit consumption had a weak inverse, instead of positive, association with levels of blood glucose, overall and in those with previously diagnosed diabetes. This is largely consistent with previous findings showing that fresh fruit consumption had no significant negative impact on glycaemic control, even in people with diabetes [5,34,39,40]. More importantly, fruit is a good source of dietary fibre [41,42], minerals (e.g., potassium [43]), and antioxidants (e.g., vitamins [44] and polyphenols [45]), which may work synergistically to confer several benefits on metabolism—including anti-oxidative, anti-inflammatory, anti-proliferative, anti-platelet, anti-hypertensive, anti-dyslipidaemic, anti-hyperglycaemic, and anti-atherogenic effects—and modulation of the composition and metabolic activity of gut microbiota [46–48], which could reduce the risk of diabetes as well as of vascular complications among those who have already developed diabetes [46,49].

In summary, our study demonstrated that, among Chinese adults, higher fresh fruit consumption was associated with lower risk of diabetes and diabetic vascular complications. Contrary to the common belief in China and many other low- and middle-income countries, fresh fruit consumption was not associated with an elevated blood glucose level in the present study, even in people with diabetes. These findings have public health and clinical implications and provide strong evidence in support of current dietary guidelines that fresh fruit consumption should be recommended for all, including those with diabetes [50]. In many developed countries, diabetes patients usually have higher fruit consumption than individuals without diabetes due to targeted health promotion and nutrition education [12,51]. However, in China people with previously diagnosed diabetes have a much lower level of fruit consumption, as observed in the present study, because of the incorrect belief that diabetes, or “sugar urine disease” in Chinese, will be better controlled if all sweet-tasting (or sugar-containing) foods, including fresh fruit, are restricted or avoided [5,52–54]. This situation emphasises the importance, given the present study findings, of better health promotion to improve public understanding of the role of fresh fruit in diabetes prevention and management. Such actions are urgently needed in China and other Asian countries, where diabetes prevalence is high and where, at the same time, there is widespread misunderstanding about eating fresh fruit among people with diabetes [40,55].

## Supporting information

S1 Fig Adjusted hazard ratios for incident diabetes associated with one daily portion of fresh fruit in subgroups of participants without diabetes at baseline. (PDF)

S2 Fig Adjusted hazard ratios for all-cause mortality and micro- and macrovascular complications of diabetes associated with one daily portion of fresh fruit in subgroups of participants with diabetes at baseline. (PDF)

S3 Fig Adjusted hazard ratios for all-cause mortality and micro- and macrovascular complications of diabetes associated with one daily portion of fresh fruit in subgroups of participants by diabetes-stage-related characteristics. (PDF)

S1 Table Baseline and usual portions per month for each baseline category of fruit consumption based on 19,788 participants who attended the first resurvey in 2008. (PDF)

S2 Table ICD-10 codes for study outcomes. (PDF)

S3 Table Baseline characteristics of participants by frequency of fresh fruit consumption at baseline (*n =* 512,891). (PDF)

S4 Table Characteristics of participants with previously diagnosed diabetes at baseline (*n* = 16,162). (PDF)

S5 Table Hazard ratios and 95% CIs (top versus bottom categories) from sensitivity analyses. (PDF)

S1 Text Original analysis plan and modifications following comments from editors and reviewers. (DOCX)

S2 Text STROBE checklist. (DOC)

## Abbreviations

BMI: body mass index
CVD: cardiovascular disease
EPIC: European Prospective Investigation into Cancer and Nutrition
HR: hazard ratio
IHD: ischaemic heart disease
RBG: random blood glucose
