# Supplementary material for: Fresh fruit consumption in relation to incident diabetes and diabetic vascular complications: A 7-y prospective study of 0.5 million Chinese adults
Source: PLoS Med. 2017 Apr 11;14(4):e1002279. doi: 10.1371/journal.pmed.1002279 (PMC5388466; doi:10.1371/journal.pmed.1002279)
Supplement: S1 Fig — (PDF) [file pmed.1002279.s001.pdf]

**S1 Fig. Adjusted HRs for incident diabetes associated with 1 daily portion of fresh fruit in subgroups of participants without diabetes at baseline**

| Subgroup | No. of events | Hazard Ratio (95% CI) |
|---|---|---|
| **Sex** | | |
| Men | 3573 | 0.90 (0.79 - 1.01) |
| Women | 5931 | 0.87 (0.79 - 0.96) |
| | | Heterogeneity test: $\chi^2_1 = 0.1$ (p=0.7) |
| **Age at risk (years)** | | |
| 35-54 | 2898 | 0.92 (0.81 - 1.06) |
| 55-64 | 3598 | 0.88 (0.77 - 0.99) |
| 65-79 | 3008 | 0.84 (0.73 - 0.96) |
| | | Trend test: $\chi^2_1 = 1.0$ (p=0.3) |
| **Area** | | |
| Urban | 3908 | 0.86 (0.78 - 0.95) |
| Rural | 5596 | 0.91 (0.81 - 1.02) |
| | | Heterogeneity test: $\chi^2_1 = 0.5$ (p=0.5) |
| **Smoking** | | |
| Never regular | 6529 | 0.86 (0.79 - 0.95) |
| Ex-regular | 737 | 0.92 (0.71 - 1.19) |
| Current regular | 2238 | 0.91 (0.78 - 1.07) |
| | | Heterogeneity test: $\chi^2_2 = 0.5$ (p=0.8) |
| **Alcohol intake** | | |
| Never regular | 8032 | 0.89 (0.82 - 0.97) |
| Ex-regular | 217 | 1.01 (0.61 - 1.68) |
| Current regular | 1255 | 0.84 (0.68 - 1.02) |
| | | Heterogeneity test: $\chi^2_2 = 0.6$ (p=0.7) |
| **Total physical activity** | | |
| 1st tertile | 3526 | 0.86 (0.76 - 0.96) |
| 2nd tertile | 3070 | 0.87 (0.76 - 1.00) |
| 3rd tertile | 2908 | 0.97 (0.84 - 1.13) |
| | | Trend test: $\chi^2_1 = 1.6$ (p=0.2) |
| **Family history of diabetes** | | |
| No | 8501 | 0.87 (0.80 - 0.94) |
| Yes | 1003 | 0.94 (0.77 - 1.16) |
| | | Heterogeneity test: $\chi^2_1 = 0.6$ (p=0.5) |
| **CVD status** | | |
| No | 7669 | 0.89 (0.82 - 0.97) |
| Yes* | 1835 | 0.87 (0.74 - 1.02) |
| | | Heterogeneity test: $\chi^2_1 = 0.1$ (p=0.8) |
| **BMI ($kg/m^2$)**** | | |
| <24 | 3235 | 0.94 (0.81 - 1.08) |
| 24-27.9 | 4088 | 0.81 (0.72 - 0.90) |
| >=28 | 2181 | 0.93 (0.80 - 1.07) |
| | | Trend test: $\chi^2_1 = 0.0$ (p=0.9) |
| **Random blood glucose (mmol/L)**** | | |
| <5.5 | 2584 | 0.90 (0.77 - 1.04) |
| 5.5-6.9 | 4116 | 0.83 (0.74 - 0.93) |
| >=7.0 | 2385 | 0.97 (0.83 - 1.12) |
| | | Trend test: $\chi^2_1 = 0.5$ (p=0.5) |
| **SBP (mmHg)** | | |
| <120 | 1642 | 0.90 (0.75 - 1.08) |
| 120-139 | 3573 | 0.93 (0.82 - 1.05) |
| >=140 | 4289 | 0.85 (0.76 - 0.96) |
| | | Trend test: $\chi^2_1 = 0.6$ (p=0.5) |
| **Season of recruitment** | | |
| Spring | 2573 | 0.87 (0.75 - 1.00) |
| Summer | 2321 | 0.93 (0.80 - 1.08) |
| Autumn | 2756 | 0.91 (0.79 - 1.05) |
| Winter | 1854 | 0.84 (0.71 - 1.01) |
| | | Heterogeneity test: $\chi^2_3 = 0.8$ (p=0.8) |
| **Overall[1]** | **9504** | **0.88 (0.81 - 0.95)** |
| **Overall[2]** | **9504** | **0.93 (0.89 - 0.97)** |

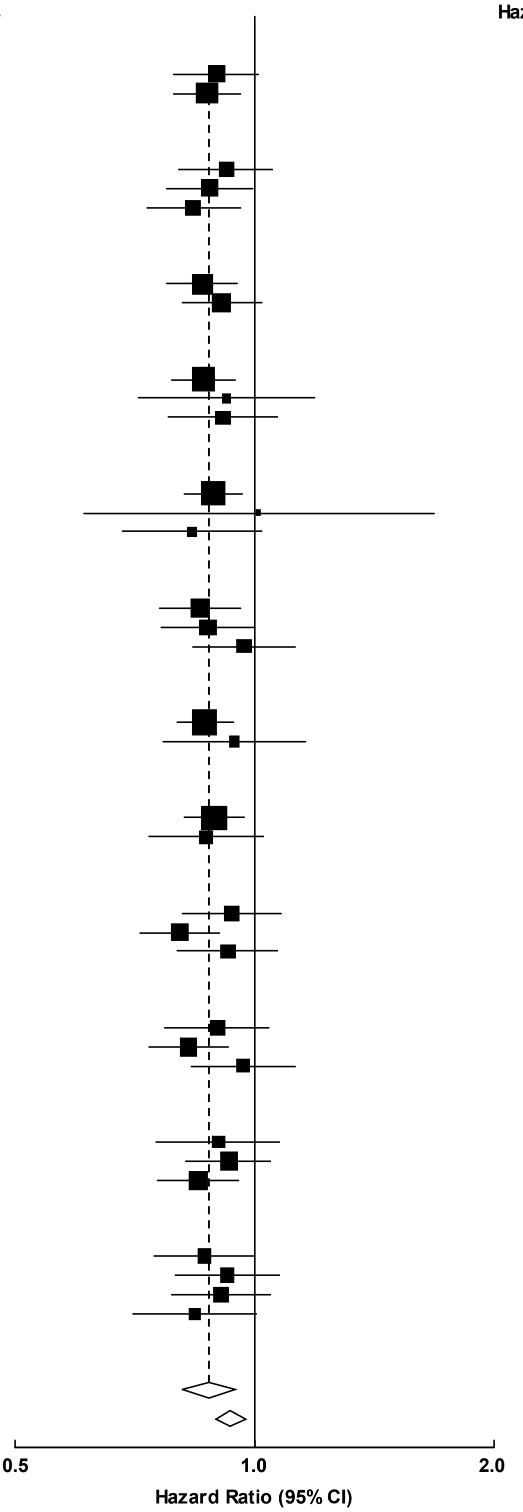


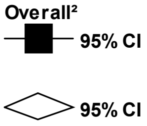


1. Overall effect of usual fresh fruit consumption after adjusting for regression dilution bias;

2. Overall effect of baseline fresh fruit consumption before adjusting for regression dilution bias.

* Including participants with either prevalent or incident CVD;

**8159 participants had missing values on blood glucose. 2 participants had missing values on BMI.
