# Supplementary material for: Fresh fruit consumption in relation to incident diabetes and diabetic vascular complications: A 7-y prospective study of 0.5 million Chinese adults
Source: PLoS Med. 2017 Apr 11;14(4):e1002279. doi: 10.1371/journal.pmed.1002279 (PMC5388466; doi:10.1371/journal.pmed.1002279)
Supplement: S3 Fig — (PDF) [file pmed.1002279.s003.pdf]

S3 Fig. Adjusted HRs for all-cause mortality and micro-and macro-vascular complications of diabetes associated with 1 daily portion of fresh fruit in subgroups of participants by diabetes-stage-related characteristics

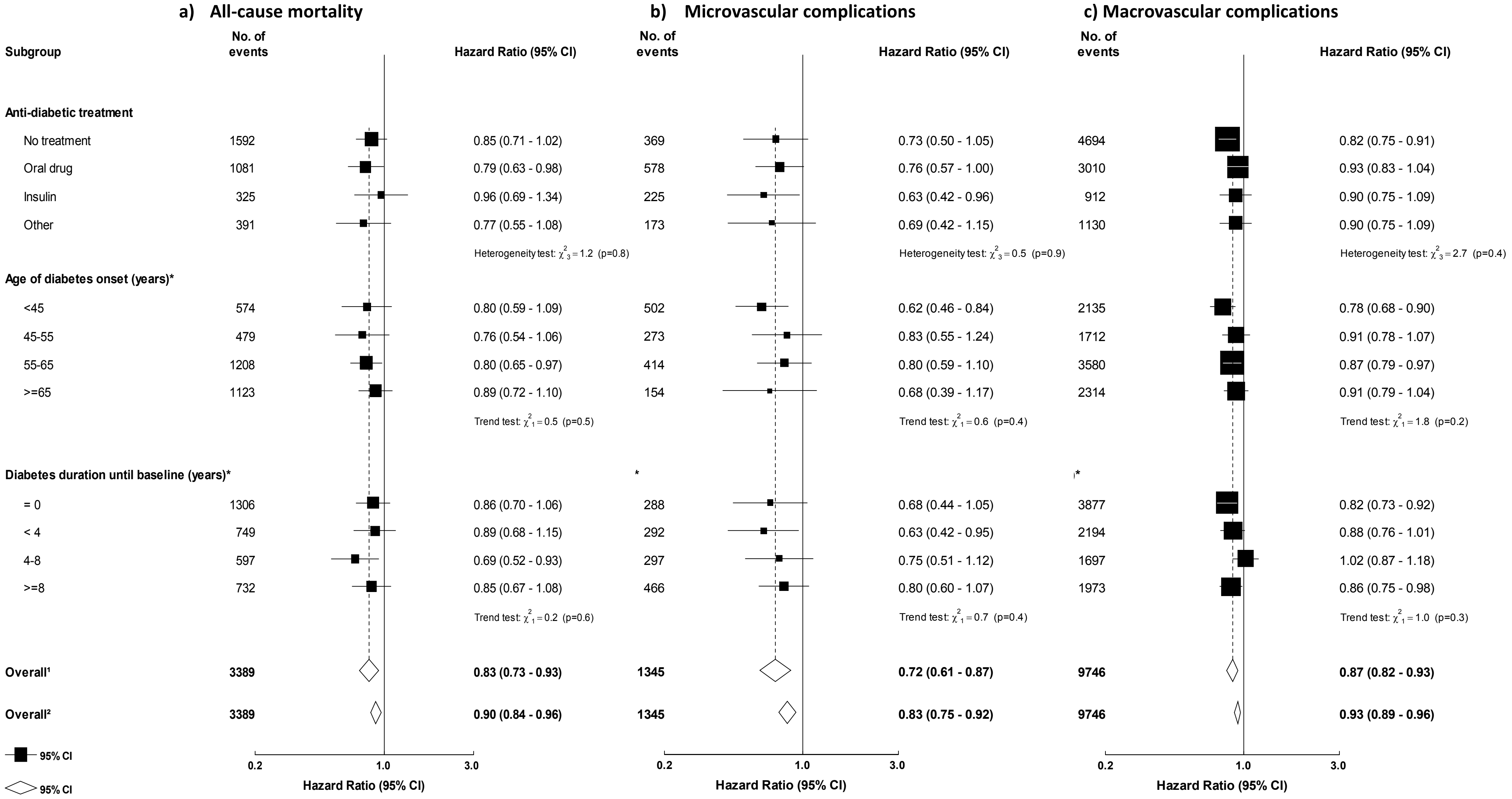

1. Overall effect of usual fresh fruit consumption after adjusting for regression dilution bias; 2. Overall effect of baseline fresh fruit consumption before adjusting for regression dilution bias.

* 21 participants had missing values on age of diabetes diagnosis and therefore diabetes duration.
