# Supplementary material for: Fresh fruit consumption in relation to incident diabetes and diabetic vascular complications: A 7-y prospective study of 0.5 million Chinese adults
Source: PLoS Med. 2017 Apr 11;14(4):e1002279. doi: 10.1371/journal.pmed.1002279 (PMC5388466; doi:10.1371/journal.pmed.1002279)
Supplement: S1 Table — (PDF) [file pmed.1002279.s004.pdf]

S1 Table. Baseline and usual portions per month for each baseline category of fruit consumption based on 19,788 participants who attended the first resurvey in 2008

	Baseline	1st resurvey (mean 2.6 years later) F					Consumption days per month	Mean daily portion*	Baseline consumption (portions/month) B	Usual consumption (portions/month)† U
	n	Never/rarely 1	Monthly 2	1-3 days/wk 3	4-6 days/wk 4	Daily 5				
1	Never/rarely	17.85 % (244)	55.74% (762)	18.00% (246)	4.24% (58)	4.17% (57)	0	-	$B_1 = 0$	$U_1 = 7.47$
2	Monthly	7.37% (496)	52.07% (3504)	29.01% (1952)	6.85% (461)	4.70% (316)	2.5	1.37	$B_2 = 3.4$ (2.5×1.37)	$U_2 = 9.74$
3	1-3 days/wk	4.00% (250)	31.70% (1981)	37.43% (2339)	11.67% (729)	15.20% (950)	8.6	1.42	$B_3 = 12.2$ (8.6×1.42)	$U_3 = 16.82$
4	4-6 days/wk	1.59% (29)	20.12% (368)	35.05% (641)	15.04% (275)	28.21% (516)	21.5	1.40	$B_4 = 30.1$ (21.5×1.40)	$U_4 = 23.71$
5	Daily	1.25% (45)	6.81% (246)	18.32% (662)	13.17% (476)	60.46% (2185)	30	1.68	$B_5 = 50.4$ (30×1.68)	$U_5 = 36.90$

*The mean daily portion number came from the 2nd resurvey data, used as a proxy of baseline mean daily portion assuming that the daily portions of fresh fruit consumption did not vary much from the baseline to the 2nd resurvey (i.e. people may change their frequency of fruit consumption but the daily amount of fresh fruit consumption remains roughly the same).

†Usual intake amount for each group was estimated by taking into account changes in consumption frequency between baseline and 1st resurvey using this formula $Un = \sum_{i=1}^{5}(Fni \times Bi)$; F is the percentage in each cell, B is the baseline proportion per month for each baseline category, U is the usual proportion per month for each baseline category.
