# Supplementary material for: Fresh fruit consumption in relation to incident diabetes and diabetic vascular complications: A 7-y prospective study of 0.5 million Chinese adults
Source: PLoS Med. 2017 Apr 11;14(4):e1002279. doi: 10.1371/journal.pmed.1002279 (PMC5388466; doi:10.1371/journal.pmed.1002279)
Supplement: S2 Table — (PDF) [file pmed.1002279.s005.pdf]

**S2 Table: ICD-10 codes for study outcomes**

| Outcomes | ICD-10 codes |
|---|---|
| Diabetes | E10-E14 |
| Total cardiovascular disease | I00-I25, I27-I88, and I95-I99 |
| Ischemic heart disease | I20-I25 |
| Stroke | I60, I61, I63 and I64 |
| Other macrovascular diseases | I00-I19, I27-I59, I62, I65-I88, I95-I99, E10.5, E11.5, E12.5, E13.5 and E14.5 |
| Diabetic nephropathy | E10.2, E11.2, E12.2, E13.2, and E14.2 |
| Diabetic retinopathy | E10.3, E11.3, E12.3, E13.3 and E14.3, but excluding diabetic cataract, diabetic glaucoma and diabetic iritis |
| Diabetic neuropathy | E10.4, E11.4, E12.4, E13.4, and E14.4 |
