# Supplementary material for: Fresh fruit consumption in relation to incident diabetes and diabetic vascular complications: A 7-y prospective study of 0.5 million Chinese adults
Source: PLoS Med. 2017 Apr 11;14(4):e1002279. doi: 10.1371/journal.pmed.1002279 (PMC5388466; doi:10.1371/journal.pmed.1002279)
Supplement: S3 Table — (PDF) [file pmed.1002279.s006.pdf]

S3 Table. Baseline characteristics of participants by frequencies of fresh fruit consumption at baseline (n=512,891)

	Consumption frequency of fresh fruit				
	**Never/rarely** (32,775)	**Monthly** (174,195)	**1-3 days/wk** (161,353)	**4-6 days/wk** (47,982)	**Daily** (96,586)
**Mean age (SD), years**	54.7 (10.7)	52.7 (11.2)	50.9 (10.6)	49.9 (10.6)	50.3 (11.9)
**Women, %**	48.0	55.8	56.7	61.9	71.0
**Urban resident, %**	31.1	22.2	43.2	54.6	84.3
**Highest education, %**					
No formal education	29.0	23.4	18.1	14.3	9.2
Primary school	34.2	35.1	33.1	29.9	26.1
Middle school or higher	36.8	41.5	48.8	55.8	64.7
**Household income, %**					
Low	44.5	36.0	27.3	18.2	15.3
Moderate	26.7	28.9	31.3	30.6	25.6
High	28.8	35.1	41.4	51.2	59.1
**Smoking, %**					
Non smokers	55.5	63.1	68.9	73.5	74.8
Ex-smokers	4.8	5.2	6.1	6.7	7.1
Current smokers	39.7	31.7	25.0	19.8	18.1
**Alcohol drinking, %**					
Non drinkers	76.9	81.9	84.1	86.0	85.7
Ex-drinkers	2.1	1.7	1.7	1.9	2.0
Current drinkers	21.0	16.4	14.2	12.1	12.3
**Physical activity (SD), MET-hr/day**	20.8 (12.7)	21.6 (12.7)	21.2 (12.0)	20.6 (12.0)	20.3 (13.4)
**BMI (SD), kg/m^2 †**	23.3 (3.3)	23.5 (3.5)	23.8 (3.3)	23.9 (3.3)	23.9 (3.7)
**SBP (SD), mmHg**	133.2 (20.0)	131.7 (21.0)	131.3 (19.8)	130.4 (19.9)	129.3 (22.2)
**Regular food consumption*, %**					
Meat	39.0	39.1	47.2	57.9	59.0
Dairy products	7.8	6.1	9.3	16.6	25.8
Preserved vegetables	27.4	21.8	21.7	24.2	23.1
Fresh vegetables	93.0	91.2	95.9	97.6	98.5
**Random blood glucose (SD), mmol/L†**	6.5 (2.3)	6.1 (2.4)	6.0 (2.3)	6.0 (2.3)	5.9 (2.6)
**Baseline diabetes status**					
Previously diagnosed diabetes	9.4	3.4	2.7	2.1	1.9
Screen-detected diabetes	2.8	2.9	2.8	2.8	2.5

Values were adjusted for age, sex, and study area where appropriate.

*Regular consumption means consuming food products for at least 4 days per week, except for fresh vegetables, which means daily consumption;

† Level of random blood glucose was missing for 8341 participants. BMI was missing for 2 participants.
