# Supplementary material for: Fresh fruit consumption in relation to incident diabetes and diabetic vascular complications: A 7-y prospective study of 0.5 million Chinese adults
Source: PLoS Med. 2017 Apr 11;14(4):e1002279. doi: 10.1371/journal.pmed.1002279 (PMC5388466; doi:10.1371/journal.pmed.1002279)
Supplement: S4 Table — (PDF) [file pmed.1002279.s007.pdf]

S4 Table. Characteristics of participants with previously diagnosed diabetes at baseline (n = 16,162)

	Consumption frequency of fresh fruit					
	**Never/rarely** (n=2893)	**Monthly** (n=4553)	**1-3 days/wk** (n=4327)	**4-6 days/wk** (n=1106)	**Daily** (n=3283)	**Overall** (n=16,162)
**Age, sex and region-adjusted**						
BMI (SD), kg/m^2	24.8 (3.4)	24.8 (3.5)	25.0 (3.4)	25.2 (3.4)	25.1 (3.5)	24.9 (3.5)
SBP (SD), mmHg	142.7 (22.2)	142.8 (22.5)	141.5 (21.8)	141.1 (21.9)	140.5 (22.8)	141.8 (22.5)
Random blood glucose (SD), mmol/L*	11.7 (5.7)	11.5 (5.8)	11.3 (5.6)	11.0 (5.6)	11.1 (5.9)	11.4 (5.7)
Anti-diabetic treatment, %	90.0	84.6	83.1	80.9	83.3	84.6
Type of treatment, %						
Oral anti-diabetic drug†	62.5	61.0	63.7	64.7	61.6	62.4
Insulin	16.7	15.0	13.2	11.6	14.3	14.5
Other treatment	20.8	24.0	23.1	23.6	24.1	23.1
Age of diabetes onset (SD), years‡	53.2 (5.4)	53.3 (5.5)	53.5 (5.4)	53.5 (5.4)	53.6 (5.6)	53.4 (9.3)
Diabetes duration (SD), years‡	5.9 (5.3)	5.8 (5.3)	5.6 (5.2)	5.6 (5.2)	5.6 (5.4)	5.7 (5.3)
**Fully adjusted ¶**						
BMI (SD), kg/m^2	24.8 (3.5)	24.8 (3.5)	25.0 (3.4)	25.1 (3.4)	25.1 (3.6)	24.9 (3.5)
SBP (SD), mmHg	142.3 (22.2)	142.4 (22.3)	141.6 (21.4)	141.6 (21.5)	141.0 (22.9)	141.8 (22.5)
Random blood glucose (SD), mmol/L*	11.6 (5.5)	11.5 (5.6)	11.3 (5.3)	11.2 (5.4)	11.2 (5.8)	11.4 (5.7)

Values are either percentage or mean (SD)

* Level of random blood glucose was missing for 181 participants.

† Use of either chlorpropamide or metaformin

‡ was missing for 21 participants.

¶ Adjusted for age, sex, region, study area, education, annual household income, smoking, alcohol intake, physical activity, consumption of meat, dairy products and preserved vegetables, survey season, family history of diabetes, anti-diabetic treatment, and, for blood glucose only, and fasting time.
