# Supplementary material for: Fresh fruit consumption in relation to incident diabetes and diabetic vascular complications: A 7-y prospective study of 0.5 million Chinese adults
Source: PLoS Med. 2017 Apr 11;14(4):e1002279. doi: 10.1371/journal.pmed.1002279 (PMC5388466; doi:10.1371/journal.pmed.1002279)
Supplement: S5 Table — (PDF) [file pmed.1002279.s008.pdf]

S5 Table. HRs and 95% CIs (top vs bottom categories) from sensitivity analyses.

	Among those without diabetes at baseline (n = 482,591)	Among those with diabetes at baseline (n = 30,300)		
	Diabetes incidence	Total mortality	Microvascular complications	Macrovascular complications
Exclude first 2 years of follow-up	0.91 (0.95-0.96)	0.85 (0.77-0.93)	0.77 (0.67-0.88)	0.94 (0.89-0.99)
Exclude prevalent CVD cases	0.89 (0.83-0.94)	0.86 (0.78-0.94)	0.73 (0.63-0.83)	0.90 (0.85-0.94)
Exclude incident myocardial infarction and stroke	0.88 (0.83-0.94)	-	-	-
+ Rice	0.87 (0.83-0.93)	0.87 (0.80-0.94)	0.75 (0.66-0.84)	0.90 (0.86-0.94)
+ Wheat	0.88 (0.83-0.94)	0.87 (0.80-0.94)	0.75 (0.67-0.85)	0.90 (0.87-0.94)
+ Other staple foods	0.89 (0.84-0.94)	0.87 (0.80-0.94)	0.74 (0.66-0.84)	090 (0.86-0.94)
+ Fresh vegetables	0.87 (0.82-0.93)	0.86 (0.80-0.94)	0.74 (0.66-0.84)	0.90 (0.86-0.94)
+ Poultry	0.87 (0.82-0.93)	0.87 (0.81-0.95)	0.74 (0.66-0.84)	0.91 (0.87-0.94)
+ Fish	0.87 (0.82-0.92)	0.88 (0.81-0.95)	0.74 (0.66-0.84)	0.91 (0.87-0.94)
+ Eggs	0.87 (0.83-0.93)	0.87 (0.80-0.94)	0.74 (0.66-0.84)	0.91 (0.87-0.95)
+ Soybean	0.87 (0.82-0.92)	0.87 (0.80-0.94)	0.75 (0.66-0.84)	0.90 (0.86-0.94)
+ Spicy food	0.88 (0.83-0.93)	0.87 (0.80-0.94)	0.75 (0.66-0.84)	0.90 (0.86-0.94)
+ Tea consumption	0.88 (0.83-0.93)	0.87 (0.80-0.94)	0.74 (0.66-0.84)	0.90 (0.86-0.94)

*For the analyses on diabetes incidence, both baseline prevalent and incident CVD were excluded; but for the analyses on diabetes complications, only prevalent CVD cases were excluded from the analyses.
