# Supplementary material for: Fresh fruit consumption in relation to incident diabetes and diabetic vascular complications: A 7-y prospective study of 0.5 million Chinese adults
Source: PLoS Med. 2017 Apr 11;14(4):e1002279. doi: 10.1371/journal.pmed.1002279 (PMC5388466; doi:10.1371/journal.pmed.1002279)
Supplement: S1 Text — (DOCX) [file pmed.1002279.s009.docx]

# S1 Text. Original analysis plan and modifications following comments from editors and reviewers

***Main objective:*** To investigate the associations of fresh fruit consumption with diabetes incidence and, among those who had baseline prevalent diabetes, diabetes-related vascular complications.

***Participants:*** All CKB participants (n=512,891) will be included.

- For the analyses of diabetes incidence, prevalent diabetes cases will be excluded, leaving 482,591 in the main analyses.
- For the analyses of diabetes complications, 30,300 participants who had either self-reported previously diagnosed diabetes (n=16,162) or newly detected diabetes (through baseline random or fasting blood glucose measurements, n=14,138) at the baseline will be included.

***Exposure:*** Self-reported fresh fruit consumption, collected at baseline through questionnaire survey (same variable collected during the first and second resurvey will also be used when correcting for regression dilution bias for the linear associations)

***Outcomes:*** Collected through linkages with death and disease registries as well as health insurance database.

- Diabetes incidence: Among those participants who were free of diabetes at baseline, all reported diabetes cases during follow-up which occurred between age 35 and 79 years will be included.
- Diabetes complications (fatal and non-fatal):
- Mortality: Overall and cause-specific mortality, including deaths with an underlying cause of cardiovascular disease (CVD), diabetes, or other.
- Vascular complications (both fatal and non-fatal):
  - Microvascular: diabetic nephropathy, retinopathy and neuropathy
  - Macrovascular complications: ischemic heart disease, stroke and other macrovascular diseases.

***Statistical analyses:*** Cox proportional hazards model.

- Proportional hazard assumption needs to be checked before using it.
- Floating absolute risk method will be used to facilitate comparisons between different groups of exposure.
- Analyses investigating associations per 1 daily portion of fresh fruit will be corrected for regression dilution bias using data from 2 resurveys.

***Covariates:*** Selected based on literature and a priori knowledge about our data (resulting from previous work examining the association of fresh fruit consumption with CVD risk):

- Age, sex, and study area will be stratified using strata statement.
- Socio-economic status (SES): education and household income.
- Lifestyle variables: smoking, alcohol consumption, physical activity, BMI (including both BMI and physical activity simultaneously is important as that will be roughly equivalent to adjusting for total energy intake).
- Consumption of red meat, dairy products, and preserved vegetables (which is used as a proxy of salt consumption). All these three are closely correlated with SES so adjusting for them would help to further control potential residual confounding from SES.
- Family history of diabetes and survey season (in order to address the potential influence of seasonality on self-reported levels of fruit consumption).
- For the analyses investigating diabetes complications, baseline status of CVD (yes/no) and diabetes (pre-diagnosed/screen-detected), and anti-diabetic treatment (no treatment, oral drugs, insulin or other medications) will also need to be taken into account.

***Sensitivity analyses:*** In order to examine the potential influence of reverse causality bias and the robustness of the findings, the following sensitivity analyses need to be considered:

- Exclude the first 2 years of follow-up.
- Exclude baseline prevalent cases of ischemic heart disease (IHD) and stroke.
- Additionally adjust for other dietary variables, e.g. rice, wheat, other staple foods, poultry, eggs, fish, fresh vegetables and soybean.
- For the analyses of diabetes incidence, it is necessary to investigate the potential influence of CVD and diabetes co-incidence. Therefore sensitivity analysis will be performed excluding incident cases of IHD and stroke.

***Subgroup analyses:*** In order to identify potential effect modifiers, the following subgroup analyses will be conducted:

- By sex, age-at-risk, region, smoking, alcohol intake, level of physical activity, family history of diabetes, CVD status, BMI, blood glucose, blood pressure and season of recruitment.
- For the analyses investigating diabetes complications, subgroup analyses by baseline diabetes status (pre-diagnosed or screen-detected) and by diabetes-stage-related characteristics such as use of anti-diabetic treatment, age of diabetes onset, and duration of diabetes will also be undertaken.

***Modification based on the comments from editors and reviewers:*** Following the suggestion of reviewers, we performed additional analyses including changing BMI from a continuous covariate to a categorical variable; additionally adjusting for blood pressure; and excluding preserved vegetables from the models. Following the editor’s request, incidence rates and 5-year risk of main study outcomes were calculated.
