# Supplementary material for: Fresh fruit consumption in relation to incident diabetes and diabetic vascular complications: A 7-y prospective study of 0.5 million Chinese adults
Source: PLoS Med. 2017 Apr 11;14(4):e1002279. doi: 10.1371/journal.pmed.1002279 (PMC5388466; doi:10.1371/journal.pmed.1002279)
Supplement: S2 Text — (DOC) [file pmed.1002279.s010.doc]

STROBE Statement—Checklist of items that should be included in reports of ***cohort studies***

|  | Item No | Recommendation | Response |
| --- | --- | --- | --- |
| **Title and abstract** | 1 | (*a*) Indicate the study’s design with a commonly used term in the title or the abstract | Done. The study’s design is indicated in the manuscript title. |
|  |  | (*b*) Provide in the abstract an informative and balanced summary of what was done and what was found | Done (in the Methods and Findings section of the abstract) |
| Introduction | | |  |
| Background/rationale | 2 | Explain the scientific background and rationale for the investigation being reported | The scientific background and rationale for the study are described in the first 2 paragraphs of the Introduction. |
| Objectives | 3 | State specific objectives, including any pre-specified hypotheses | The objectives of the study are described in the third paragraph of the Introduction. |
| Methods | | |  |
| Study design | 4 | Present key elements of study design early in the paper | These are presented in the Methods section. Including, for example, **“*CKB is a large nationwide prospective cohort study involving 10 diverse regional sites in China, chosen to cover a wide range of risk exposures and disease patterns.”*** and ***“All participants were followed up (2411 [0.5%] participants were lost to follow-up by 1^st^ January 2014) for death and disease using information collected through linkages with death and disease registries and health insurance databases.”*** |
| Setting | 5 | Describe the setting, locations, and relevant dates, including periods of recruitment, exposure, follow-up, and data collection | The setting, locations, relevant dates, and periods of recruitment are reported in the “Study population” section of the Methods (***e.g. “10 diverse regional sites in China”*** and ***“between June 2004 and July 2008”***. Exposure is described in the “Data collection” section of the Methods: “***At the local study clinics, trained health workers administered a laptop-based questionnaire on socio-demographic status, smoking, drinking, diet, physical activity, and medical history; measured anthropometrics and blood pressure***”. Methods of follow-up were described in the “Follow-up for mortality and morbidity” section of the Methods (see above the response to item 4). |
| Participants | 6 | (*a*) Give the eligibility criteria, and the sources and methods of selection of participants. Describe methods of follow-up | The eligibility criteria, and the sources and methods of selection of participants are provided in the “Study population” section of the Methods (***i.e. “all non-disabled permanent residents aged 35 to 74 were invited to participate in the study”.***  Methods of follow-up are described in the “Follow-up for mortality and morbidity” section of the Methods (see above the response to item 4) |
|  |  | (*b*) For matched studies, give matching criteria and number of exposed and unexposed | N/A |
| Variables | 7 | Clearly define all outcomes, exposures, predictors, potential confounders, and effect modifiers. Give diagnostic criteria, if applicable | Done. The exposure (fresh fruit consumption) is defined in the “Data collection” section: “***At the local study clinics, trained health workers administered a laptop-based questionnaire on socio-demographic status, smoking, drinking, diet, physical activity, and medical history***” and “***Dietary data covered 12 major food groups, including fresh fruit, fresh and preserved vegetables, meat and dairy products, each with 5 frequency levels about habitual consumption during the past 12 months (daily, 4-6 days/week, 1-3 days/week, monthly, or never/rarely)***”.  Outcomes are defined in the “Follow-up for mortality and morbidity” section of the Methods: (***e.g. “incident diabetes included all reported cases (fatal or not) of new onset diabetes which occurred between age 35 and 79 years”, “Diabetes-related microvascular complications included nephropathy, retinopathy, and neuropathy. Macrovascular complications included IHD, stroke and other” and “Underlying causes of deaths were classified as diabetes, cardiovascular disease (CVD), or other”.***  Predictors, potential confounders and effect modifiers are defined in the “Statistical analysis” section of the Methods. e.g. “***… hazard ratios (HRs) and 95% CIs for diabetes incidence by fresh fruit consumption were estimated using Cox proportional hazard regression, adjusting for education (4 categories), annual household income (4 categories), smoking (4 categories), alcohol intake (4 categories), physical activity (continuous variable), BMI (continuous variable), consumption of meat (3 categories), dairy products (3 categories) and preserved vegetables (5 categories), survey season (4 categories), and family history of diabetes (dichotomous)”***. ***“Screen-detected diabetes was defined as having never been diagnosed with diabetes, but having a measured RBG level ≥7.0 mm****o****l/L with time since last food/beverages ≥8 hours, or ≥11.1 mmol/L with time since last food/beverages <8 hours, or a FBG level ≥7.0 mmol/L on subsequent testing.”*** |
| Data sources/ measurement | 8* | For each variable of interest, give sources of data and details of methods of assessment (measurement). Describe comparability of assessment methods if there is more than one group | Such information is provided in the “Data collection” section of the Methods, e.g. “***The blood glucose levels were measured immediately following sample collection using the SureStep Plus System (Johnson and Johnson, USA), …***”. |
| Bias | 9 | Describe any efforts to address potential sources of bias | As described in various places in the Methods, recruitment was approximately random, limiting selection bias. We have corrected for regression dilution bias which may result from measurement errors and long-term variation in the exposure. Outcomes were derived from multiple different data sources, limiting biases inherent in the use of a single data source, and coding of disease events was blinded, further reducing the risk of bias. In sensitivity analyses, we excluded the first 2 years of follow-up in order to limit the potential of reverse causality. |
| Study size | 10 | Explain how the study size was arrived at | Described in the “Study population” and “Statistical analyses” sections of the Methods, including “***all non-disabled permanent residents aged 35 to 74 were invited to participate in the study, and about one in three (33% in rural areas, 27% in urban) responded, involving a total of 512,891 participants (including a few who were just outside the targeted age range)”, “Among 482,591 participants who were free of diabetes at baseline,…” and “Among those 30,300 participants with diabetes at baseline, …”***. |
| Quantitative variables | 11 | Explain how quantitative variables were handled in the analyses. If applicable, describe which groupings were chosen and why | Described in the “Statistical analyses” section of the Methods, see above the response to item 7. |
| Statistical methods | 12 | (*a*) Describe all statistical methods, including those used to control for confounding | The statistical methods used are described throughout the “Statistical analyses” section of the Methods. |
|  |  | (*b*) Describe any methods used to examine subgroups and interactions | Described at the end of “Statistical analyses” section of the Methods, e.g. ***“Adjusted HRs for each 1 daily portion of usual fruit consumption were calculated across strata of potential effect modifiers e.g. diabetes risk and diabetes-stage related factors, and chi-square tests for trend and heterogeneity were applied to the log HRs and their standard errors.”*** |
|  |  | (*c*) Explain how missing data were addressed | There were no missing data for the exposure and outcome variables. Missing data for other variables are described for simple descriptive analyses in Table 1’s legend and in the footnotes of SFigs. For the main analyses, 2 participants with missing data for BMI were automatically excluded from the Cox model analyses. |
|  |  | (*d*) If applicable, explain how loss to follow-up was addressed | Loss to follow-up is described in the “Follow-up for morbidity and mortality” section of the Methods: “***2411 (0.5%) were lost to follow-up by 1st January 2014***.” Those who were lost to follow-up were censored on the date of loss to follow-up. |
|  |  | (*e*) Describe any sensitivity analyses | Sensitivity analyses are described in the last paragraph of “Statistical analyses” section of the Methods, e.g. ***“sensitivity analyses investigated the potential impacts of excluding the first 2 years of follow-up, excluding participants with prevalent CVD at baseline and with additional adjustment for other dietary factors”.*** |
| Results | | |  |
| Participants | 13* | (a) Report numbers of individuals at each stage of study—eg numbers potentially eligible, examined for eligibility, confirmed eligible, included in the study, completing follow-up, and analysed | Done. See above the response to item 10. |
|  |  | (b) Give reasons for non-participation at each stage | NA |
|  |  | (c) Consider use of a flow diagram | - |
| Descriptive data | 14* | (a) Give characteristics of study participants (eg demographic, clinical, social) and information on exposures and potential confounders | Characteristics of study participants are presented in Table 1 and in the first and second paragraphs of the Results section: e.g. “***Of the 512,891 participants, 30,300 (5.9%) had diabetes at baseline, including 16,162 with previously-diagnosed diabetes and 14,138 with screen-detected diabetes***” “***Individuals with diabetes were older, more likely to be women, to live in urban areas, less physical active and had higher levels of BMI, waist circumference and blood pressure. Among men, the proportions of current regular smoking and alcohol drinking were about 10% lower in previously diagnosed diabetes than in screen-detected diabetes or those without diabetes.”*** |
|  |  | (b) Indicate number of participants with missing data for each variable of interest | The number of participants with missing data for each variable is presented in the Table 1 legend and the footnotes of SFigs 1-3. |
|  |  | (c) Summarise follow-up time (eg, average and total amount) | Follow-up time is summarised in the third paragraph of the Results section: “***During ~3.4 million person-years (mean 7 years) of follow-up***…” |
| Outcome data | 15* | Report numbers of outcome events or summary measures over time | The numbers of outcome events are presented in the text, all figures and Table 3. |
| Main results | 16 | (*a*) Give unadjusted estimates and, if applicable, confounder-adjusted estimates and their precision (eg, 95% confidence interval). Make clear which confounders were adjusted for and why they were included | Adjusted HRs and 95% confidence intervals are provided and covariates were mentioned in the footnotes of Table 3 and legends of all figures. |
|  |  | (*b*) Report category boundaries when continuous variables were categorized | NA |
|  |  | (*c*) If relevant, consider translating estimates of relative risk into absolute risk for a meaningful time period | Table 2 contains incidence rates for the main outcomes. |
| Other analyses | 17 | Report other analyses done—eg analyses of subgroups and interactions, and sensitivity analyses | Findings of multiple subgroup and sensitivity analyses are presented throughout the Results section, in S5 Table and S1-3 Fig. |
| Discussion | | |  |
| Key results | 18 | Summarise key results with reference to study objectives | The key results are summarised in the first paragraph of the Discussion: “***This large prospective study of adult Chinese with and without diabetes showed that higher fresh fruit consumption was significantly associated with a lower risk of developing diabetes and also a lower risk of dying or developing vascular complications among those who have already developed diabetes. These associations appeared to be similar in both men and women, in urban and rural residents, in those with previously diagnosed and screen-detected diabetes. Moreover, higher fresh fruit consumption was not associated with an elevated level of blood glucose***.” |
| Limitations | 19 | Discuss limitations of the study, taking into account sources of potential bias or imprecision. Discuss both direction and magnitude of any potential bias | Limitations of the study are described in the Discussion, e.g.: “***However, the study also has limitations. … Finally, although we have carefully adjusted for potential confounders and there were consistent results across different participant subgroups, residual confounding (e.g. by socio-economic status) may still persist***.” |
| Interpretation | 20 | Give a cautious overall interpretation of results considering objectives, limitations, multiplicity of analyses, results from similar studies, and other relevant evidence | A cautious overall conclusion has been given in the last paragraph of the Discussion. |
| Generalisability | 21 | Discuss the generalisability (external validity) of the study results | The generalisability of the study results to the Chinese population as a whole and to non-Chinese populations is discussed in the last the paragraph of the Discussion section. |
| Other information | | |  |
| Funding | 22 | Give the source of funding and the role of the funders for the present study and, if applicable, for the original study on which the present article is based | Done. |

*Give information separately for exposed and unexposed groups.

**Note:** An Explanation and Elaboration article discusses each checklist item and gives methodological background and published examples of transparent reporting. The STROBE checklist is best used in conjunction with this article (freely available on the Web sites of PLoS Medicine at http://www.plosmedicine.org/, Annals of Internal Medicine at http://www.annals.org/, and Epidemiology at http://www.epidem.com/). Information on the STROBE Initiative is available at http://www.strobe-statement.org.
